# Spontaneous bacterial peritonitis caused by *Campylobacter Coli* in cirrhotic patient: A rare case report (CARE-compliant)

**DOI:** 10.1097/MD.0000000000019887

**Published:** 2020-05-22

**Authors:** Cheng-Hui Wang, Ting-Han Tai, Shih-Yen Weng, Shin-Wen Yeh, Sheng-Jie Shiue, Ganbolor Jargalsaikhan, Ming-Shun Wu

**Affiliations:** a Department of Laboratory Medicine, Wan Fang Hospital; b School of Medical Laboratory Science and Technology; c Division of Gastroenterology, Department of Internal Medicine, Wan Fang Hospital, Taipei Medical University, Taipei, Taiwan; d International PhD program in Medicine, College of Medicine, Taipei Medical University; e Division of Gastroenterology and Hepatology, Department of Internal Medicine, School of Medicine, College of Medicine, Taipei Medical University, Taipei; f Research Center for Healthcare Industry Innovation, National Taipei University of Nursing and Health Sciences; g Integrative Therapy Center for Gastroenterologic Cancers, Wan Fang Hospital, Taipei Medical University, Taipei; h Department of Primary Care Medicine, Shuang Ho Hospital; i Department of Orthopedics, Shuang Ho Hospital, Taipei Medical University, New Taipei City, Taiwan.

**Keywords:** *campylobacter Coli*, mALDI-TOF, peritonitis, sBP

## Abstract

**Introduction:**

Spontaneous bacterial peritonitis (SBP) is a fatal infection in patients. It often happens in patients with cirrhosis, cancer or diabetes, and is caused mostly by *Enterobacteriaceae*. Here we report a rare case of SBP caused by *Campylobacter Coli* (*C coli*) infection, which was identified promptly by the matrix assisted laser desorption ionization-time of flight mass spectrometry (MALDI-TOF MS) and received adequate therapy sooner after.

**Patient concerns:**

In the present study, we reported a 46-year-old male with alcoholic liver cirrhosis (Child-Pugh class C) and type 2 diabetes mellitus presented with a 1-day history of fever and abdominal pain.

**Diagnosis:**

Based on the clinical examinations, the patient was diagnosed with SBP and the pathogen was quickly identified as *C coli* by the matrix assisted laser desorption ionization-time of flight mass spectrometry (MALDI-TOF MS), a rare causative pathogen of SBP.

**Interventions:**

The patient received a 10-day antibiotic treatment with Ciprofloxacin 400 mg every 12 hours, and recovered successfully.

**Outcomes:**

The patient had a successful treatment outcome.

**Conclusion:**

The study demonstrated a new possible infectious cause of SBP by *C Coli*, which was rarely seen in liver cirrhosis but mostly found in immunocompromised patients. Thus, it might raise an idea of microorganism screening of broader types that might also induce SBP for immunocompromised patients.

## 1 Introduction

Spontaneous bacterial peritonitis (SBP), which is a fatal bacterial infection commonly accompanied with liver cirrhosis characterized by portal hypertension and splanchnic vasodilation, leads to the imbalance of sodium and water that cause the ascites retention in the peritoneal cavity. SBP is often caused by *Enterobacteriaceae* including Gram-negative bacilli such as *E coli* and *Klebsiella*.^[1]^

*Campylobacter coli (C coli),* a spiral microaerophilic gram-negative bacillus, is known to cause sepsis in immunocompromised patients, which is highly prevalent in patients with cancer, liver cirrhosis, or diabetes.^[2]^ If elderly people, children and immunocompromised patients get infected with *C Coli*, it can be fatal.^[3]^ Here we reported a rare case of SBP caused by *C coli*, which was only described in a previous case report published in 1987.^[4]^ In this case report, we also discussed the epidemiology, clinical features, evaluation, and treatment of *C coli* infections.

## 2 Case presentation

A 46-year-old male with medical history of alcoholic liver cirrhosis (Child-Pugh class C) for 3 years and type 2 diabetes mellitus around 10 years presented a one-day history of fever and abdominal pain. His abdomen was distended with shifting dullness. Laboratory examination showed the white blood cell (WBC) count was 8500 cell/μl with neutrophils of 86.50%, random glucose was 384 mg/dl (reference range 70–140 mg/dl), Cr was 1.04 mg/dl (reference range 0.7–1.3 mg/dl), total bilirubin was 6.60 mg/dl (reference range 0.1–1.4 mg/dl), ALT was 27 U/l (reference range < 20 U/l), ALP was 114 U/l (reference range 34–104 U/l), GGT was 195 U/l (reference range 9–64 g/dl) and CRP was 1.9 mg/dl (reference range < 0.5 mg/dl). Ascites fluid obtained through paracentesis revealed the WBC count of 743 cell/μl with 79% of neutrophils. Patient had a smoking history of one pack per day for 30 years and drank 16 bottles of beer per week for 20 years.

**Figure 1:**
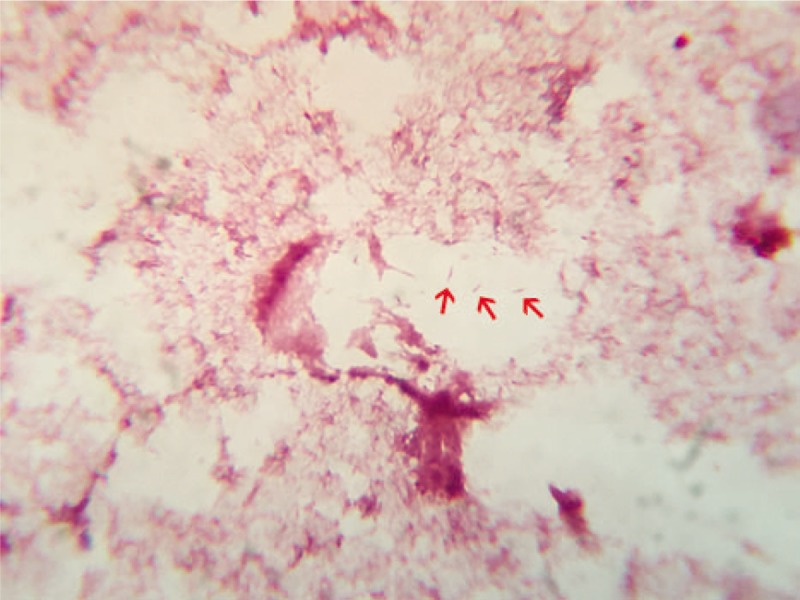
Gram stain of the blood culture isolate showed spiral Gram-negative and spiral bacilli (arrows).

**Figure 2:**
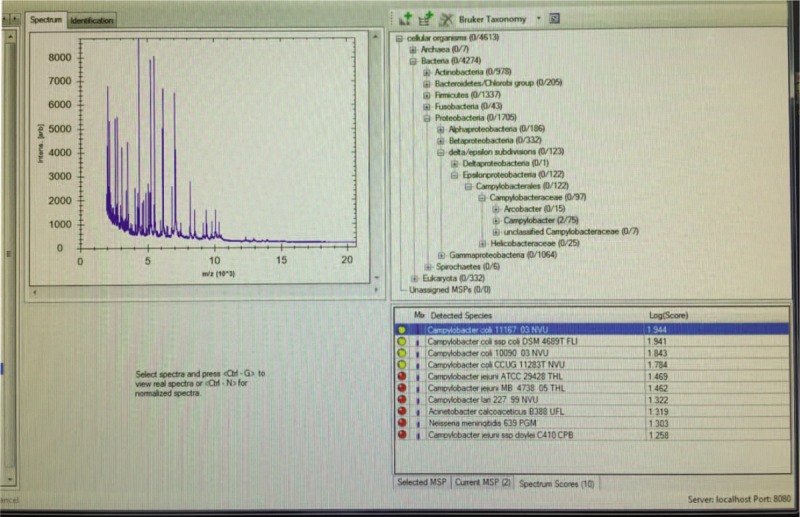
Typical mass spectra of (left panel) and the scoring and taxonomy system (right panel) for the identification of *C Coli*. The scores ranged from 1.784 to 1.944, namely the pathogen had high similarity with *C coli*.

Based on the patient's history, physical examination, and paracentesis results, he was diagnosed with SBP. Blood cultures were positive for Gram-negative spiral bacilli (Fig. 1). *C coli* was further identified by using the matrix assisted laser desorption ionization-time of flight mass spectrometry (MALDI-TOF MS, Bruker IVD MALDI Biotyper). According to the standard interpretative criteria recommended by the manufacturer, the specie cutoff value was ≥2.000, the genus cutoff value was 1.700–1.999, and the non-identifiable score values was ≤ 1.699. The log scores ranged from 1.784 to 1.944 (Fig. 2). Our MALDI-TOF MS results showed that the pathogen had high identity to *C coli*. The patient immediately received a 10-day course of intravenous ciprofloxacin 400 mg every 12 hours instead of Cefotaxime, the most commonly used antibiotics for SBP, and had a successful treatment outcome.

## 3 Discussion

SBP caused by *Campylobacter* is rare. The first case report of SBP caused by *Campylobacter* was presented by Targan et al in 1976. Since then, 5 cases have been reported, 3 of which were associated with peritonitis and bacteremia.^[4]^ The main underlying conditions of these patients were liver-related disease, including alcohol abuse, cirrhosis, cancer, diabetes, and chemotherapy. In accordance with previous studies, the patient from our case report was at a higher risk for *Campylobacter* infection for his severe alcoholic liver cirrhosis and diabetes mellitus.^[5,6]^ However, the identification of *Campylobacter* species is laborious due to their complexity in taxonomy. Traditional identification method by PCR amplification of gene locus from 16S rRNA and 18S rRNA is not accurate and time-consuming.^[7,8]^

Third generation cephalosporins are considered the standard empirical treatment for spontaneous bacterial peritonitis in people with cirrhosis. However, based on case reports in immunocompromised situation, carbapenem is warranted to eradicate *Campylobacter* followed by de-escalating strategy according to the sensitivity test of antibiotics.^[9]^ In critical circumstances, serum bactericidal activity against *Campylobacter* may decrease in the immunocompromised patients with hypogammaglobulinemia. In previous study, *Campylobacter* was susceptible to ampicillin, ceftriaxone, erythromycin, ciprofloxacin, gentamicin and imipenem.^[6]^ In our case report, the patient with *C coli septicemia* recovered sooner after administration of intravenous Ciprofloxacin. Nevertheless, treatment with antibiotics alone may fail to eradicate the pathogen. Combination therapy with immunoglobulin and antibiotics should be considered to treat such patients.^[10]^

To improve the method of identification, MALDI-TOF MS is applied for phenotyping featured by cell-extract proteins representing specific bacteria.^[11]^ The new method is relatively faster and more sensitive for the identifying processes such as strain typing, epidemiological studies, antibiotic resistance, etc.^[12]^ To our knowledge, this is the first report of *C coli*-related SBP confirmed by MALDI-TOF MS. It is imperative to have a strong doubt about this infection, particularly for the immunocompromised population. Therefore, our results explore a novel method for the diagnosis of SBP, which largely reduce the time and cost and improve the accuracy of traditional methods.

## Author contributions

**Conceptualization:** Ming-Shun Wu.

**Data curation:** Cheng-Hui Wang, Ting-Han Tai, Shin-Wen Yeh.

**Formal analysis:** Ting-Han Tai.

**Investigation:** Cheng-Hui Wang, Ting-Han Tai, Shin-Wen Yeh, Ming-Shun Wu.

**Project administration:** Ming-Shun Wu.

**Validation:** Cheng-Hui Wang, Ting-Han Tai, Shin-Wen Yeh.

**Writing – original draft:** Ming-Shun Wu.

**Writing – review & editing:** Shih-Yen Weng, Sheng-Jie Shiue, Ganbolor Jargalsaikhan, Ming-Shun Wu.

## References

[1] Dever JB Sheikh MY Review article: spontaneous bacterial peritonitis – bacteriology, diagnosis, treatment, risk factors and prevention. Aliment Pharmacol Ther 2015;41:1116–31. 25819304 10.1111/apt.13172

[2] Pacanowski J Lalande V Lacombe K Campylobacter Bacteremia: Clinical Features and Factors Associated with Fatal Outcome. Clin Infect Dis 2008;47:790–6. 18699745 10.1086/591530

[3] Sheppard SK Maiden MC The evolution of Campylobacter jejuni and Campylobacter coli. Cold Spring Harbor Perspect Biol 2015;7:a018119. 10.1101/cshperspect.a018119 PMC4526750 26101080

[4] Ho H Zuckerman MJ Polly SM Spontaneous bacterial peritonitis due to Campylobacter coli. Gastroenterology 1987;92:2024–5. 3569775 10.1016/0016-5085(87)90639-1

[5] Yoon JG Lee SN Hyun HJ Campylobacter jejuni Bacteremia in a Liver Cirrhosis Patient and Review of Literature: A Case Study. Infect Chemother 2017;49:230–5. 28608661 10.3947/ic.2017.49.3.230 PMC5620392

[6] Hadano Y Iwata H An unusual cause of spontaneous bacterial peritonitis due to Campylobacter fetus with alcoholic liver cirrhosis. BMJ Case Rep 2013;2013:bcr2012008406. 10.1136/bcr-2012-008406 PMC3603790 23417384

[7] Müller W Böhland C Methner U Detection and genotypic differentiation of Campylobacter jejuni and Campylobacter coli strains from laying hens by multiplex PCR and fla-typing. Res Veterin Sci 2011;91:e48–52. 10.1016/j.rvsc.2011.01.028 21349563

[8] Liu YH Yamazaki W Huang YT Clinical and microbiological characteristics of patients with bacteremia caused by Campylobacter species with an emphasis on the subspecies of C. fetus. J Microbiol Immunol Infect 2019;52:122–31. 28801089 10.1016/j.jmii.2017.07.009

[9] Chavez-Tapia NC Soares-Weiser K Brezis M Antibiotics for spontaneous bacterial peritonitis in cirrhotic patients. Cochrane Database Syst Rev 2009. 10.1002/14651858.CD002232.pub2 PMC7100568 19160207

[10] Allos BM Iovine NM Blaser MJ Bennett JE Dolin R Blaser MJ 218 - Campylobacter jejuni and Related Species. Mandell, Douglas, and Bennett's Principles and Practice of Infectious Diseases (Eighth Edition). Philadelphia: Content Repository Only; 2015 2485–2493.e2484.

[11] Jang K-S Kim YHJJoM Rapid and robust MALDI-TOF MS techniques for microbial identification: a brief overview of their diverse applications. J Microbiol 2018;56:209–16. 29492868 10.1007/s12275-018-7457-0

[12] Singhal N Kumar M Kanaujia PK MALDI-TOF mass spectrometry: an emerging technology for microbial identification and diagnosis. Front Microbiol 2015;6: 10.3389/fmicb.2015.00791 PMC4525378 26300860

